# Ultrasound analysis of different forms of hemolytic uremic syndrome in children

**DOI:** 10.3389/fped.2024.1433812

**Published:** 2024-10-23

**Authors:** Lydia Rink, Ilja Finkelberg, Martin Kreuzer, Lukas Schipper, Lars Pape, Metin Cetiner

**Affiliations:** ^1^ Children’s Hospital, Pediatrics II, Pediatric Nephrology, University of Essen, Essen, Germany; ^2^ Institute for Medical Informatics, Biometry and Epidemiology, University of Duisburg-Essen, Essen, Germany

**Keywords:** hemolytic uremic syndrome, acute kidney failure, complement activation, dialysis, ultrasound, renal size, resistance index, children

## Abstract

**Background:**

Hemolytic uremic syndrome (HUS) is the most common cause of acute kidney injury in children. It is mainly caused by Shiga toxin-producing enterohemorrhagic *Escherichia coli* (EHEC; STEC-HUS) and is more rarely caused by uncontrolled complement activation (cHUS). Renal replacement therapy is frequently required and kidney function recovers in the majority of patients. Ultrasound (US) is the preferred imaging modality for the evaluation of any renal failure. The aim of this study is the evaluation of US diagnostics in both HUS types at disease onset and in the course of the disease.

**Materials and methods:**

Clinical, laboratory, and US data from the digital patient records of children admitted as inpatients with a diagnosis of HUS were recruited for a monocentric, retrospective analysis. STEC-HUS and cHUS were diagnosed when, in addition to the laboratory constellation, EHEC infection and complement system activation were verified, respectively. US examinations were performed by pediatricians with certified pediatric US experience.

**Results:**

In total, 30 children with STEC-HUS (13/25 male; median age of disease onset 2.9 years; most prevalent EHEC serotype was O157) and cHUS (2/5 male; median age of disease onset 5.4 years; 3/5 with proven pathogenic variation) were included. Renal replacement therapy proportions were comparable in the STEC-HUS and cHUS patients (64% vs. 60%). The resistance index (RI) was elevated at disease onset in the patients with STEC-HUS and cHUS (0.88 ± 0.10 vs. 0.77 ± 0.04, *p* = 0.13) and was similar in the STEC-HUS subcohorts divided based on dialysis requirement (yes: 0.86 ± 0.1; no: 0.88 ± 0.1; *p* = 0.74). Total kidney size at disease onset displayed a positive correlation with dialysis duration (R = 0.53, *p* = 0.02) and was elevated in both HUS types (177% ± 56 and 167% ± 53). It was significantly higher in the STEC-HUS subcohort which required dialysis (200.7% vs. 145%, *p* < .029), and a regressor kidney size threshold value of 141% was indicated in the receiver operating characteristic analysis. A classification model using both US parameters sequentially might be of clinical use for predicting the need for dialysis in patients with STEC-HUS. The US parameters normalized over time.

**Conclusion:**

The US parameters of RI and total kidney size are valuable for the assessment of HUS at disease onset and during therapy, and may be helpful in the assessment of whether dialysis is required in patients with STEC-HUS.

## Introduction

Hemolytic uremic syndrome (HUS) is the most common cause of acute kidney failure in children under the age of 5 years, requiring dialysis. HUS is a thrombotic microangiopathy (TMA) that appears histologically and pathophysiologically as damage to endothelial cells, the subsequent activation of platelets, and, following thrombotic occlusion of small vessels, ultimately tissue damage. The three eponymous symptoms are intravascular hemolysis, thrombocytopenia, and acute kidney injury (AKI) and they are sensitive but not specific for the diagnosis of HUS as there are numerous differential diagnoses of TMA; these are mainly thrombotic thrombocytopenic purpura (TTP), numerous autoimmune diseases, disseminated intravascular coagulation (DIC), some medications, and infections (1–4).

HUS is typically categorized as caused by infection with Shiga toxin-producing enterohemorrhagic *Escherichia coli* (EHEC; 90% of all HUS cases, “STEC-HUS”) or as complement-mediated HUS (“cHUS”), usually caused by uncontrolled complement activation, among other HUS forms (2, 4).

The released Shiga toxin in STEC-HUS causes endothelial cell damage. The most frequent age at onset of the disease is between 2 and 5 years and there is no gender difference. The most common EHEC serogroup is O157:H7 (somatic O antigen 157 and flagellar H antigen 7) (5–7). A massive HUS outbreak in Germany with over 800 HUS cases was caused by an *E. coli* serotype, O104:H4, which was distributed by contaminated sprouts, and predominantly affected adults (8, 9). The health authorities should be notified when STEC-HUS is detected.

Complement-mediated HUS is present in 5%–10% of all pediatric HUS cases with an incidence of 2 per 1,000,000. Uncontrolled activation of the alternative pathway of complement activation with the subsequent formation of C5b-9 complexes (membrane attack complex) that damages the endothelial cell surface in the kidneys and other organs represents the pathophysiological starting point for TMA (1, 10).

The majority of complement-mediated HUS cases result from heterozygous pathogenic variants in genes encoding for complement regulatory proteins factor H (CFH), factor I (CFI), factor B (CFB), and factor 3 (C3), or in the regulators membrane cofactor protein (MCP) and thrombomodulin (11). Particularly in the case of children, acquired antibodies against factor H, often combined with pathogenic changes in genes responsible for complement factor H-related proteins (CFHR), represent a further cause of complement-mediated HUS (1, 10, 12).

Among other HUS forms are the rare hereditary forms due to a pathogenic diacylglycerol kinase ɛ (DGKE) (13) or due to a cobalamin C deficiency (14). Acquired HUS is a rare complication of invasive infection with *Streptococcus pneumonia* (15) and there are secondary forms of HUS with underlying causes including allogeneic stem cell transplantation, various diseases such as systemic lupus erythematosus, and certain groups of drugs such as calcineurin inhibitors (16).

Impairment of renal function in STEC-associated HUS develops approximately 7–14 days after the onset of enteritic symptoms. The proportion of patients who need replacement therapy varies between 27% and 60% in different studies and kidney function recovers in the majority of the patients (3). The therapy in STEC-HUS is supportive and the proportion of STEC-HUS patients with permanent symptoms such as proteinuria, arterial hypertension, impaired average renal function, and extrarenal lasting symptoms amounts to approximately 30%. The acute mortality rate of STEC-HUS has been reduced to less than 5% and is mainly due to central nervous system (CNS) complications due to thrombotic microangiopathy (3, 6, 17). In contrast to complement-mediated HUS, STEC-HUS does not recur. The prognosis of complement-mediated HUS is significantly worse with higher mortality rates and more frequent progression to end-stage renal disease (ESRD) (17, 18). The prognosis improved significantly due to humanized monoclonal antibodies against complement protein C5 (eculizumab and ravalizumab) and they are therefore recommended as the first-line therapy for complement-mediated HUS (19, 20).

The laboratory constellation for microangiopathic hemolytic anemia with the presence of fragmentocytes, elevated lactate dehydrogenase (LDH) and bilirubin levels, reduced haptoglobin, a negative antiglobulin test, and frequently impaired renal function apply to all forms of HUS (2). A kidney biopsy is usually not necessary for the diagnosis of the primary forms of HUS (3). An ultrasound (US) is the preferred imaging modality for the initial evaluation of any renal failure and US information on renal morphology (size, echogenicity, and corticomedullary differentiation) and perfusion (Doppler US, flow velocity, and flow profile) supports the differentiation between acute and chronic kidney failure (21–23). US is a non-invasive technique and plays an important role in diagnostic support, therapy monitoring, and long-term follow-up in HUS (24–28).

The aim of our retrospective study was the analysis and evaluation of US diagnostics in STEC-HUS and complement-mediated HUS at the onset of the disease and during the clinical course.

## Material and methods

### Patient recruitment and data collection

**Figure 1:**
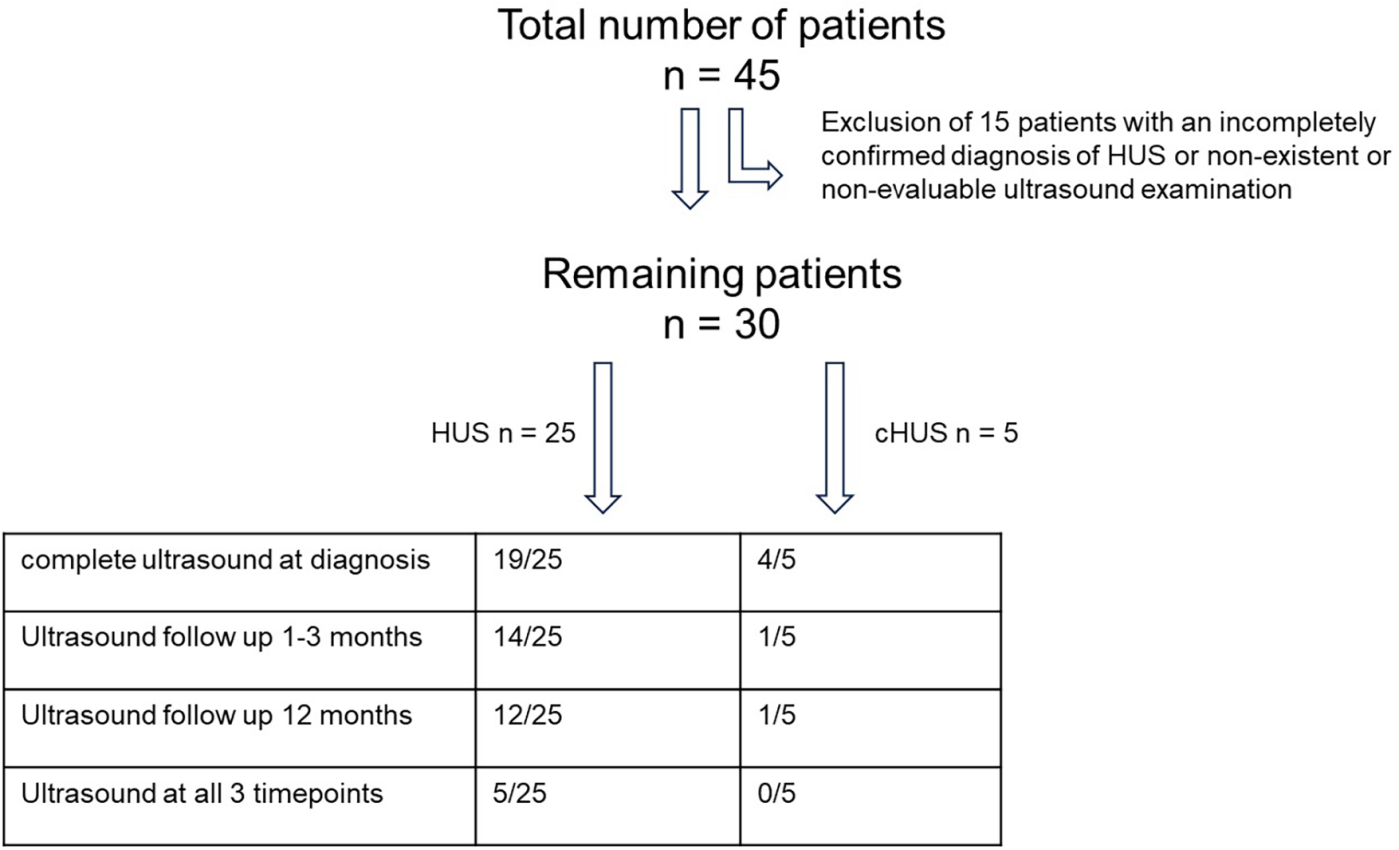
Selection algorithm for patients with HUS including ultrasound examination.

Between 2017 and 2022, 45 children were admitted as inpatients with an initial diagnosis of HUS to the Department of Pediatric Nephrology at the Children’s Hospital of the University of Duisburg-Essen, Germany. We consecutively recruited 30 of the 45 children (as shown in Figure 1) for retrospective analysis according to the following inclusion criteria. STEC-HUS was only diagnosed when there was microbiological evidence of *E. coli* infection, specifically stool cultures that were found to be positive for *E. coli* that produce Shiga toxin 2 (PCR and/or enzyme immunoassay) at the National EHEC Reference Center in Germany and/or through the detection of EHEC antibodies and when all three of the following main criteria are met: (1) thrombocytopenia, defined as a platelet count below 150,000/μl; (2) microangiopathic hemolytic anemia; and (3) acute kidney injury, defined as creatinine or cystatin c levels greater than 1.5 times the upper normal limit for that age. Complement-mediated HUS was diagnosed based on the laboratory parameters mentioned above, signs of complement system activation [including C3, C3d, C4, sC5b-9, activity of the classical (CH50) and alternative (APH50) complement pathways, and complement factor H antibody] and/or a genetic diagnosis confirmation of pathogenic variants in genes encoding for complement regulatory proteins, and, if necessary, by histopathological evidence of renal microangiopathy. Correspondingly, the exclusion criteria were defined as the absence of at least one of the aforementioned inclusion criteria and any potential clinical signs or any previous diagnoses that might cause a secondary form of HUS.

Clinical and laboratory data were collected from digital patient records. Implementation, type, and duration of a potential renal replacement therapy, supportive care, and potential therapeutic measures such as plasma exchange and drug-based complement blockade were also included in the analysis.

### Standard ultrasound examination

As part of the routine clinical assessment and standard of care, all children with suspected impairment of renal function or suspected HUS underwent an abdominal ultrasound examination upon hospital admission. Standard US examination included an assessment of kidney size; parenchymal structure, including echogenicity and corticomedullary differentiation, kidney perfusion, including systolic and diastolic flow velocities; and renal resistance index (RI) analyzed by Doppler US technology. The kidney size was measured as the length (cm). The examination also included evaluation of liver size and structure, spleen size and structure, and presence of an ascites intestinal structure with signs of colitis such as a thickened intestinal wall and increased intestinal wall perfusion. The liver size was measured in the sternal line. The dimension of the spleen was determined below the left costal margin. All organ sizes are given as a percentage of the normal age- and height-related values (29).

For better comparability of kidney size, the measured lengths were standardized to normal kidney size for that age (30). The literature refers to the percentage of kidney size in comparison to the normal size (50th percentile kidney length) for the corresponding age and sex group, rather than the total kidney size. Enlarged kidneys were defined in the normal population as kidney lengths above the 97.5th percentile. This corresponds to a percentage kidney size in relation to the 50th percentile of 114.6% ± 1.8% (minimum 112%, maximum 121.2%). Based on this, kidney sizes greater than 120% of the age-based norm were considered enlarged for this study. RI values were compared with published normal values in children and adults (31, 32).

Ultrasound examinations were performed using an Aplio i800 (Canon Medical Systems Corporation, Otawara, Japan) with an i8CX1 transducer (PVI-475BT, single curved, 1.8–6.2 MHz) and an I18LX5 transducer (PLI-1205BX, linear, 5.0–18.0 MHz) and using a Zonare ZS3 (ZONARE Medical Systems, Mountain View, CA, USA) with a single curved C6-2 transducer and a linear L14-5 transducer. The pediatricians on duty with certified pediatric US experience performed all the US examinations.

All 30 patients underwent a US kidney perfusion analysis; however, only 23 of the 30 patients had a complete renal US examination including an assessment of the renal parenchyma and their kidney size. A follow-up US examination at the time of hospital discharge or in the following 2 weeks was performed in 15 of the 30 patients. A follow-up US examination 12 months after the initial diagnosis of HUS was performed in 13 of the 30 patients. A US examination at all three timepoints was performed in 6 of the 30 patients; this was due to the fact that the patients no longer presented to our clinic for follow-up.

Please refer to Figure 1 for a flowchart of the patients and available data for analysis and Table 1 for patient characteristics. The study was conducted in accordance with the Declaration of Helsinki and approved by the institutional review board at the University Hospital in Essen. As the analysis was retrospective and used only anonymized parameters obtained for routine clinical assessments, individual written consent was not required.

**Table 1:** Clinical and laboratory findings and treatment variables during inpatient treatment.

Number	Age at diagnosis (years)	Sex	HUS	Minimal eGFR (ml/min/1.73 m^2^)	Maximum LDH (U/L)	Minimal thrombocytes (/nl)	Minimal hemoglobin (g/dl)	Dialysis (days)	Form of dialysis
1	2.4	M	STEC-HUS	15	2,902	255	6.8	17	PD
2	1.2	M	STEC-HUS	19	2,608	47	6.2	14	HD
3	0.8	M	STEC-HUS	20	1,586	81	5.5	0	
4	7.7	M	STEC-HUS	15	3,601	30	4.8	27	HD
5	6	F	STEC-HUS	17	2,777	93	5.8	26	HD
6	0.8	M	STEC-HUS	8	1,808	52	6.4	10	PD
7	2.5	F	STEC-HUS	9	1,361	83	6	5	PD
8	2.5	F	STEC-HUS	16	2,836	25	6	17	PD
9	3.1	M	STEC-HUS	12	3,065	61	4.7	4	PD
10	1.6	F	STEC-HUS	67	5,386	2	5.7	0	PD
11	1.2	F	STEC-HUS	12	2,578	56	6.3	24	PD
12	3.8	M	STEC-HUS	54	3,830	13	2.5	0	
13	1	M	STEC-HUS	12	2,467	89	7	15	PD
14	16.5	F	STEC-HUS	39	1,084	53	7.1	5	PD
15	12.8	F	STEC-HUS	9	2,455	20	5.6	6	PD
16	3.2	F	STEC-HUS	12	3,655	21	4.3	30	PD
17	0.9	F	STEC-HUS	6	959	310	5.3	0	
18	7.7	F	STEC-HUS	34	3,101	7	4.5	0	
19	2.9	M	STEC-HUS	12	4,039	29	5.1	8	PD
20	1.8	M	STEC-HUS	30	2,658	21	5.6	0	
21	2.9	M	STEC-HUS	25	1,903	63	6.5	0	
22	8.2	F	STEC-HUS	21	1,985	62	11.2	24	PD
23	14.5	F	STEC-HUS	40	1,058	48	5.8	0	
24	5.5	M	STEC-HUS	20	1,238	175	5.3	0	
25	7.6	M	cHUS	12	3,870	51	5.4	13	HD
26	11.1	F	cHUS	28	3,691	21	5.9	0	
27	3.1	F	cHUS	7	3,500	36	3.9	35	PD
28	3.8	M	cHUS	22	2,968	88	4	0	
29	5.4	F	cHUS	6	804	90	5.2	212	HD
30	2.5	M	STEC-HUS	11	3,458	70	6.8	32	PD

F, female; HD, hemodialysis; m, male; PD, peritoneal dialysis.

### Statistical analyses

Statistical analyses were performed with SPSS (version 29) for Windows (IBM Corp, Armonk, NY, USA). Groups were compared by independent *t*-test or—in the case of multiple groups—using ANOVA with Dunnett's *post-hoc* analysis; Chi-square or Fisher's exact tests were used for comparisons between groups for categorical data. The significance levels were set at 0.05 unless stated otherwise.

For the calculation of a threshold value in relation to kidney size for the classification of the requirement for dialysis in patients with HUS, we first defined the regressor variable kidney size. This was defined as the quotient of the percentage deviation of the measured kidney size of the respective patient and the 50th percentile of the age-appropriate standard value (30). Our dependent variable was the binary variable dialysis requirement: yes vs. no.

We calculated different threshold values for the regressor variable, in which patients below the respective threshold value were classified as not requiring dialysis and patients with a value of at least this threshold value were classified as requiring dialysis. The resulting receiver operating characteristic (ROC) curve reflects the different specificities and sensitivities of the various thresholds used. The optimal threshold value was selected based on the maximum Youden index value (33). Balanced accuracy is indicated by the sensitivity and specificity means and reflects the importance of both values. The area under the curve (AUC) of the ROC curve is a measure of the degree to which the kidney size is suitable as a classifier for the different threshold values (33).

Equivalently, we repeated the ROC analysis with the regressor RI value defined as the quotient of the percentage deviation of the measured RI values and the 50th percentile of the age-appropriate standard values (31).

In addition, we examined the classification capability of the two variables together using a decision tree and calculated thresholds for both of them. First, a classification was made using kidney size. Patients who were classified as requiring dialysis were then subdivided again using the RI value variable to improve the overall sensitivity and specificity compared to a classification based solely on kidney size or solely on RI value. The Gini index was used as the splitting index. For a detailed description, see Figure 4 and the corresponding caption.

For calculating the ROC in this analysis, we used R version 4.1.2 (2021-11-01) and the “roc” function of the R-package pROC. The decision tree was calculated via the “rpart” function of the R-package rpart.

## Results

### Patient characteristics

The STEC-HUS cohort included 25 children (aged 0.8–16.5 years, mean ± SD 4.8 ± 4.2 years, median 2.9 years). The distribution of sex was nearly balanced with 52% (13/25) male patients. The patients had normal weight (z-score mean ± SD, −0.4 ± 1.3, range −4.9 to 2.2) and body length (z-score mean ± SD, 0.4 ± 1.5, range −1.7 to 6.1). Their mean minimum platelet count was 70 ± 73/μl (range 2–310/μl), their mean minimum hemoglobin level was 5.8 ± 1.5 g/dl (range 2.5–11.2 g/dl), and their mean maximum LDH level was 2,576 ± 1,083 U/L (range 959–5,386 U/L). The Shiga toxin in the stool and/or EHEC was detected in all the STEC-HUS patients. The most prevalent EHEC serotype was O157 (36% of all cases), serotypes O126, O145, and O156 were each found in one patient (each correlating to 4% of all cases).

The cohort of complement-mediated HUS included five children (aged 2.2–10.2 years, mean ± SD 6.2 ± 3.2 years, median 5.4 years). The distribution of sex was nearly balanced with 40% (2/5) male patients. The patients had normal weight (z-score mean ± SD, −0.1 ± 1.1, range 1.1–1.5) and length (z-score mean ± SD, 0.2 ± 0.7, range −0.8 to 0.9). Their mean minimum platelet count was 57 ± 31/μl (range 21–90/μl), their mean minimum hemoglobin level was 4.9 ± 0.9 g/dl (range 3.9–5.9 g/dl), and their mean maximum LDH level was 2,967 ± 1,255 U/L (range 804–3,870 U/L). In three of the five patients (60%) with cHUS, the disease specific/causing pathogenic variants were known. One patient had a deletion leading to the loss of CFHR 1 and 3 causing CFH antibodies. Each patient had homozygous pathogenic variations in the CD46 gene, encoding for membrane cofactor protein, and in the C3 gene, encoding for complement protein C3. In the remaining two cHUS patients without genetic evidence, the diagnosis was confirmed by sustained complement activation and histopathological evidence of both chronic and acute thrombotic microangiopathy with arteriolar and glomerular involvement.

The individual data of the children with HUS are shown in Table 1.

The symptoms reported at hospital admission were similar in the patients with STEC-HUS and cHUS regarding seizures (16% vs. 20%) and macrohematuria (12% vs. 20%). However, diarrhea was reported more frequently in the STEC-HUS patients than in the cHUS patients (68% vs. 40%), but hematochezia was only reported in STEC-HUS patients (36%). Arterial hypertension was more frequent in cHUS than in STEC-HUS patients (40% vs. 8%) (Table 2).

**Table 2:** Accompanying clinical symptoms in patients with STEC-HUS or cHUS at disease onset.

		STEC-HUS (25)	cHUS (5)
HT	No HT	23 (92%)	3 (60%)
	Reversible HT	2 (8%)	1 (20%)
	Irreversible HT	0 (8%)	1 (20%)
Seizure	Yes	4 (16%)	1 (20%)
	No	21 (84%)	4 (80%)
Diarrhea	Yes	17 (68%)	2 (40%)
	No	9 (36%)	3 (60%)
Macrohematuria	Yes	3 (12%)	1 (20%)
	No	22 (88%)	4 (80%)

HT, hypertension.

A blood transfusion was performed in 63.3% of cases (60% in the STEC-HUS group and 80% in the cHUS group). Two patients (8%) with STEC-HUS received thrombocyte transfusions perioperatively with thrombocyte counts of 2 and 7/nl, respectively; no cHUS patient received a thrombocyte transfusion.

The complement factor C3 was reduced in 16.6% of cases (12% in the STEC-HUS group and 40% in the cHUS group).

### Renal impairment

The mean estimated glomerular filtration rate (eGFR) at the initial presentation was 21.4 ± 15.1 ml/min/1.73 m^2^ in the STEC-HUS patients and 14.7 ± 9.5 ml/min/1.73 m^2^ in the complement-mediated HUS patients.

The proportion of patients that received renal replacement therapy was similar in STEC-HUS and cHUS patients (64% vs. 60%). While the median dialysis duration was similar (STEC-HUS 10 days vs. cHUS 13 days), the mean duration of dialysis was significantly longer in the cHUS patients with 49.6 days (range 0–212 days) compared to 10.5 days (range 0–32 days) for the STEC-HUS patients (*p* ≤ 0.001). In one patient with cHUS, kidney function did not recover despite therapy with eculizumab and the patient received peritoneal dialysis for 7 months and subsequently underwent a successful kidney transplantation. Excluding this patient’s mean dialysis duration in the cHUS group lowered the overall mean duration to a comparable 12 days. All the other patients showed normalization of kidney function. In a subgroup analysis, patients with O157 serotype STEC-HUS (*n* = 9) had the lowest rate of dialysis at 55.5% (Table 3).

**Table 3:** Dialysis requirement and duration in patients with STEC-HUS or cHUS.

		STEC-HUS (16)	O157 STEC-HUS (9)	cHUS (5)
Dialysis (*n*)	Yes	10 (62.5%)	5 (55.5%)	4 (80%)
	No	6 (37.5)	4 (45.5%)	2 (20%)
Duration of dialysis (days)	Median	10	4.5	13
	Mean (standard deviation)	11.5 (11.1)	8.6 (11.7)	49.6 (85.3)
	Minimum	0	0,00	0
	Maximum	32	30,00	200
From of dialysis	HD	2 (20%)	0 (0%)	2 (50%)
	PD	8 (80%)	5 (100%)	2 (50%)
Chronic kidney failure	Yes	0 (0%)	0 (0%)	1 (20%)
	No	0 (0%)	0 (0%)	4 (80%)

Dialysis was indicated in 63.3% of cases, hemodialysis was performed in 21.1%, and peritoneal dialysis in 78.9%.

## Ultrasound parameters

### US renal size

**Figure 2:**
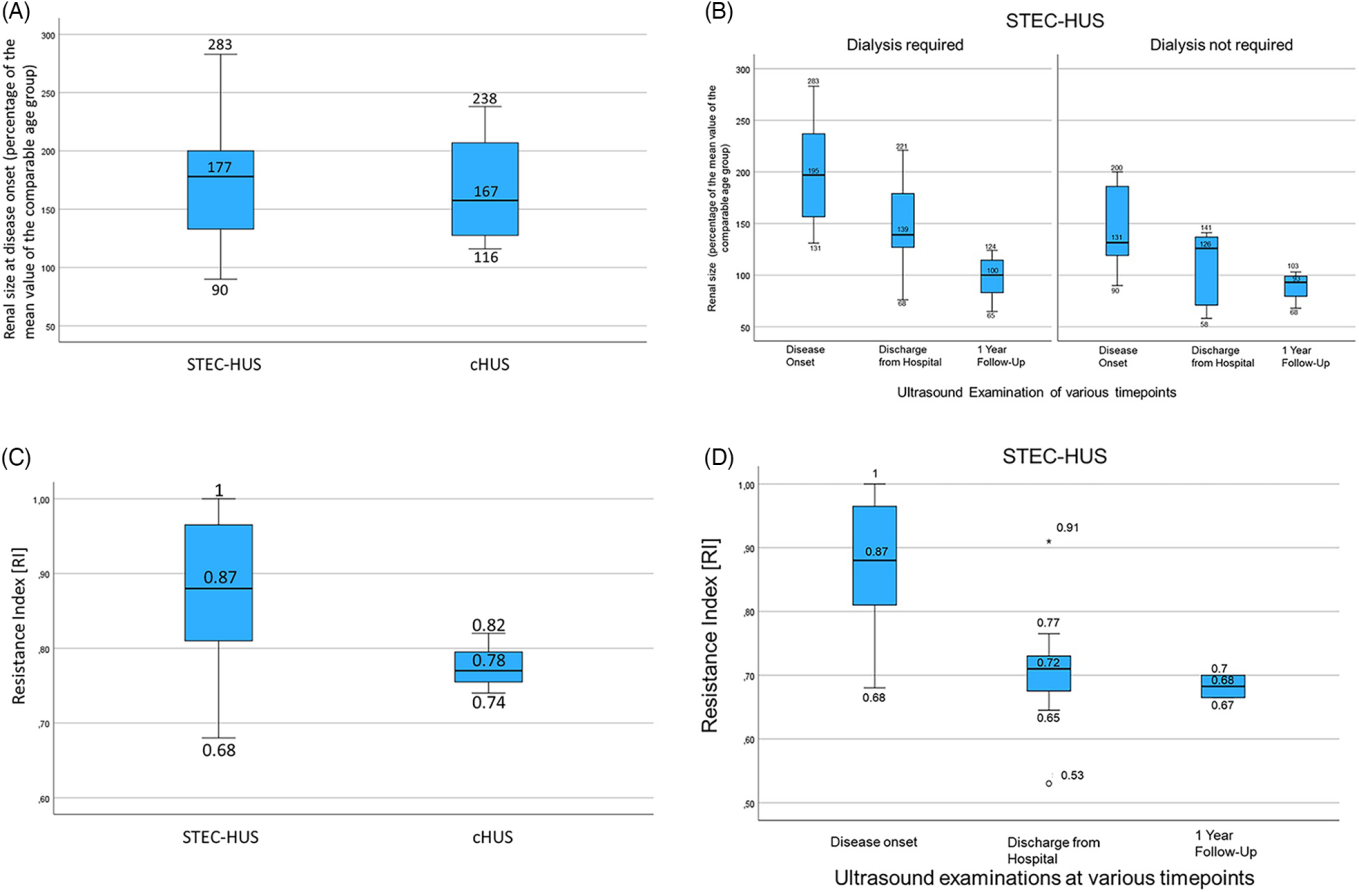
**(A)** A boxplot of age-adjusted kidney size in STEC-HUS and cHUS patients at disease onset. Both were significantly elevated compared to the normal range but not statistically significantly different from each other. **(B)** A boxplot of age-adjusted kidney size in STEC-HUS patients who required and did not require dialysis at disease onset, at the time of discharge, and at follow-up after 1 year shows significantly higher kidney sizes in STEC-HUS patients who required dialysis compared to those who did not. In both groups, kidney size returns to the age norm with clinical improvement. **(C)** A boxplot of RI in STEC-HUS and cHUS patients at disease onset shows lower RI values in the cHUS patients without demonstrable statistical significance due to the small sample size. **(D)** A boxplot of RI in the STEC-HUS patients at disease onset, recovery, and follow-up shows high values for RI at disease onset decreasing to the normal range in accordance with clinical improvement.

The total kidney size at disease onset for all patients with STEC-HUS represented as a percentage of the normal age-adjusted mean value was elevated (177.4% ± 56.2%, 95% CI: 150–204) and insignificantly higher than in the cHUS patients (167.0% ± 53.3%, 95% CI: 82–252, Figure 2A). In the subcohort of STEC-HUS patients who required renal replacement therapy, the total kidney size was significantly higher at 200.7% (±56%, 95% CI: 163%–238%) compared to the STEC-HUS cases who did not require dialysis at 145% (±40%, 95% CI: 112%–179%) (Figure 2B; *p* < 0.029; one-way ANOVA assessment). There were no outliers, according to inspection with a boxplot. Data was normally distributed for each group (Shapiro–Wilk test, *p* > 0.05) and there was homogeneity of variance (Levene's test, *p* > 0.05). While there was no correlation for the overall cohort between the kidney size at disease onset and the duration of dialysis (R = 0.18, *p* = 0.39), the subgroup analysis for the STEC-HUS patients revealed a positive correlation (R = 0.53, *p* = 0.02) for kidney size at disease onset and dialysis duration. The ROC analysis revealed a regressor kidney size threshold of 141% (1.41), for the need for dialysis in STEC-HUS patients. The corresponding sensitivity was 0.909, the specificity 0.625, and the balanced accuracy 0.767. This means that 76.7% of our patients (*N* = 19) were correctly classified. The corresponding ROC curve with an AUC of 0.8125 is shown in Supplementary Figure S1A.

The echogenicity of the renal parenchyma was increased in 76.9% of the patients (20/36) [STEC-HUS: 81.8% (18/22); cHUS 50% (2/4)].

The mean kidney size in the STEC-HUS group decreased at the time of discharge with a still slightly bigger size in the subcohort that required dialysis (146.8%; ±9.2%, 95% CI: 109–185 vs. 106.6%; ±39.1%, 95% CI: 58–155; *p* = 0.14) and it normalized in both STEC-HUS subcohorts after 1 year (98%; ±20.5%, 95% CI: 81–115 vs. 89.3%; ±15%, 95% CI: 64–115) (Figure 2B).

### Resistance index

**Figure 3:**
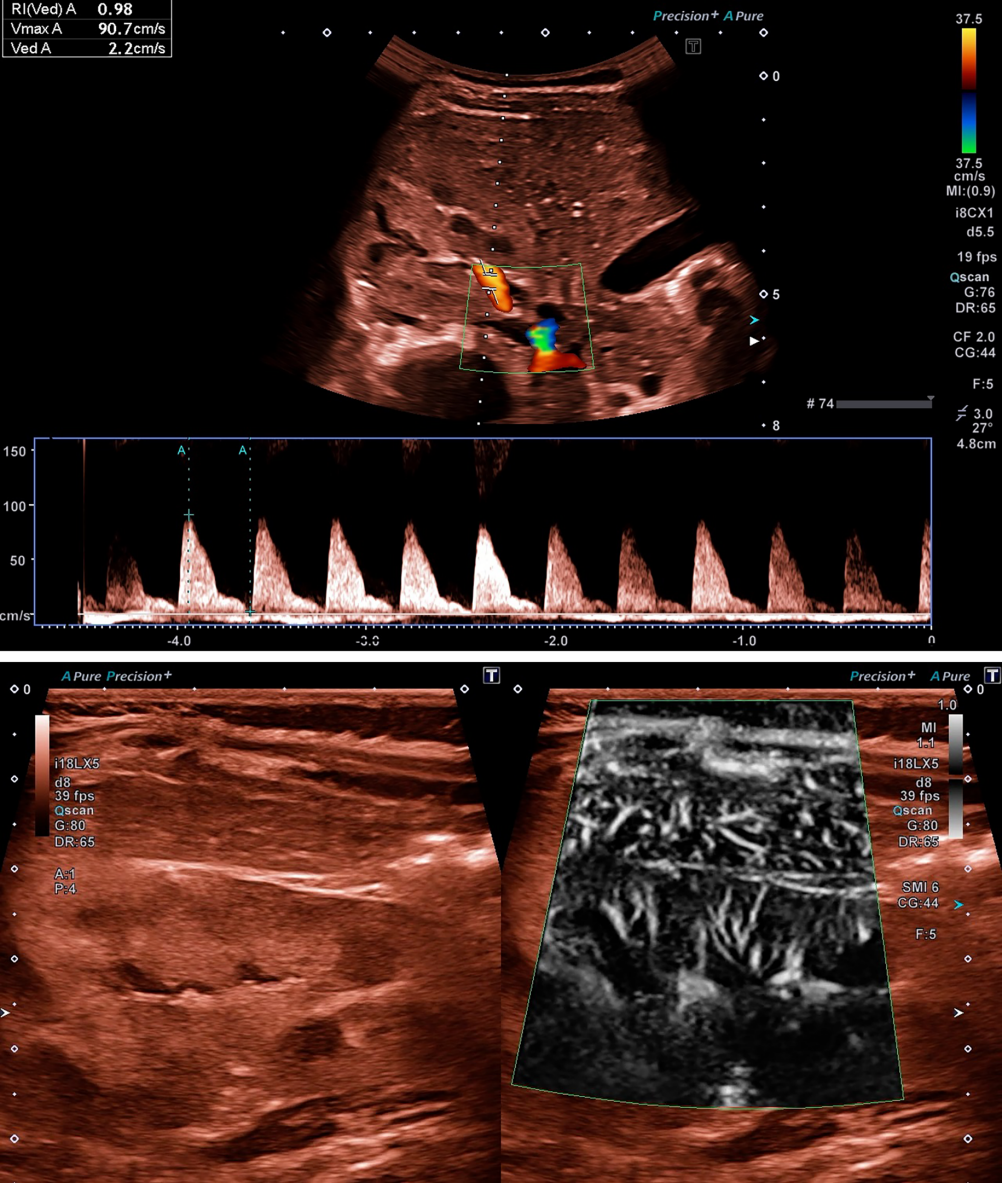
**(A)** Ultrasound examination of renal perfusion at disease onset with evidence of a significantly increased value for RI of 0.98 in a 10-month-old girl with STEC-HUS. **(B)** Reduced renal tissue perfusion in SMI mode, which is atypically even lower than the overlying hepatic tissue perfusion, in the same patient as in **(A)**. Video presentation in the Supplementary Material Video S1.

The mean RI, an important ultrasound parameter for organ perfusion, was elevated at disease onset in the STEC-HUS (0.87 ± 0.10) and cHUS patients (0.78 ± 0.04) compared to normal values. However, the difference in mean RI values between the STEC-HUS and cHUS did not reach statistical significance (*p* = 0.13) even when taking into account the small cHUS cohort (Figures 2C, 3A). The RI values at the onset of the disease were also not significantly different in the children who received a blood transfusion (RI 0.88 ± 0.09 vs. 0.82 ± 0.10; *p* = 0.20). The RI values were comparable in the STEC-HUS subcohort that required dialysis compared to those who did not (0.86 ± 0.1 vs. 0.88 ± 0.1; *p* = 0.74) and these normalized at the time of discharge and remained within the normal range 1 year later (Figure 2D). The RI and dialysis duration in days showed no correlation (R = 0.11, *p* = 0.65).

The ROC analysis revealed a regressor RI value threshold of 1.098. The corresponding sensitivity was 0.333, the specificity 0.857, and the balanced accuracy 0.595, which was only slightly better than a random estimate. The corresponding ROC curve with an AUC of 0.5119 is shown in Supplementary Figure S1B.

### Decision tree

**Figure 4:**
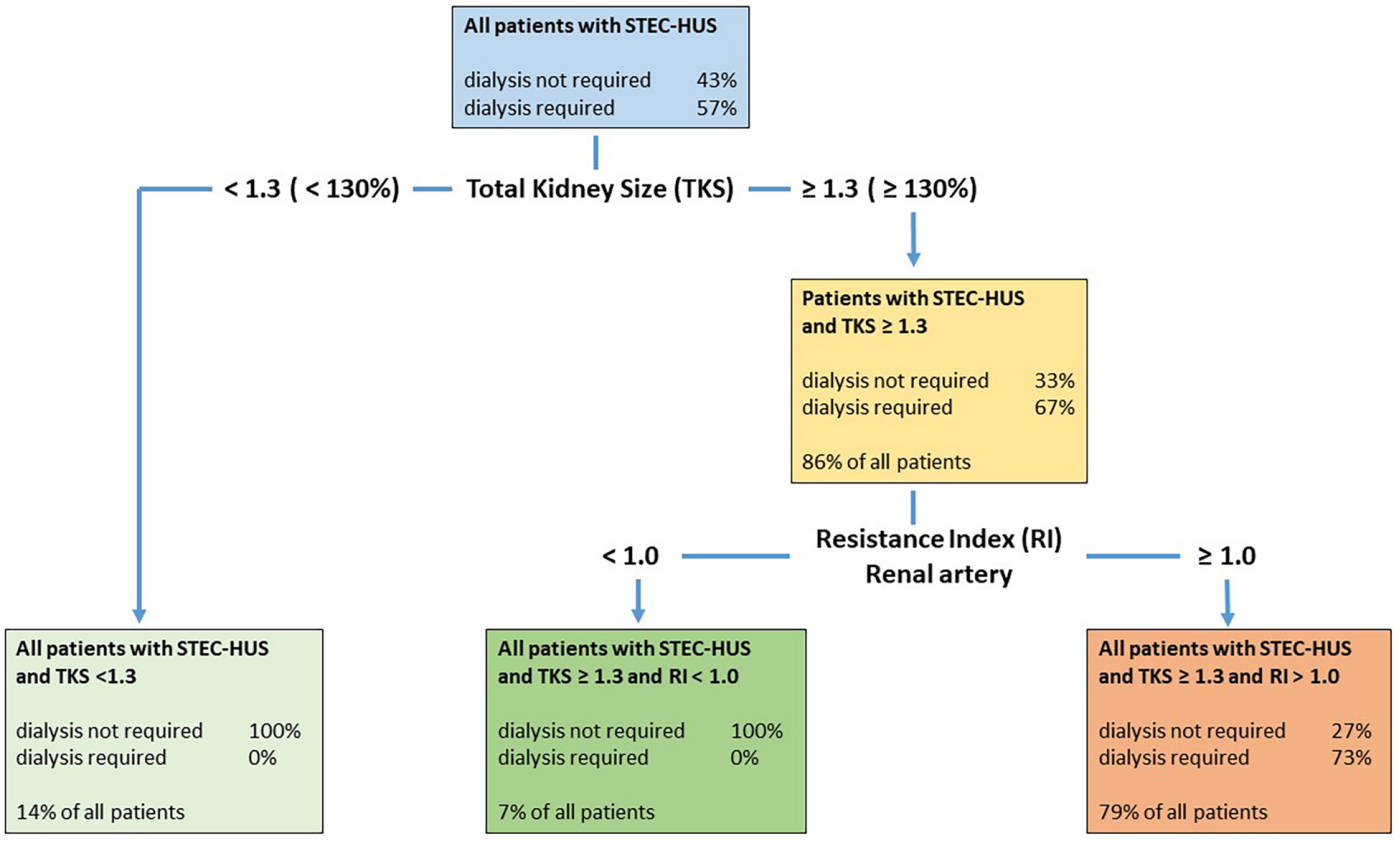
Decision tree with both classificatory variables, kidney size and RI, in patients with STEC-HUS. The decision tree first classifies the patients based on the kidney size variable and then based on the RI value variable. The algorithm used calculates suitable threshold values that optimize the classification using these variables. Patients with a total kidney size (TKS) ≥131% were classified as requiring dialysis, but only 67% actually required dialysis. Patients with an RI value ≥1.0 were classified as requiring dialysis; however, only 73% really required dialysis. This means that all patients requiring dialysis were recognized as such. However, some patients who did not require dialysis were also classified as requiring dialysis. This resulted in a sensitivity of 1.0 and a specificity of 0.7273.

For *n* = 14 patients, we calculated a threshold of 129.5% (1.295), for the variable kidney size and a threshold of 1.01 for the RI value. The corresponding sensitivity was 1.0, the specificity 0.7273, and the balanced accuracy 0.8636 (Figure 4).

### Other US parameters

In individual cases, reduced renal microperfusion due to thrombotic microangiopathy had already been visualized using the superb microvascular imaging (SMI) mode. The atypically weaker tissue perfusion of the kidney compared to the overlying liver served as a comparison here (Figure 3B).

Liver size was only available in 56.6% of patients (17/30) and there was no age-adjusted hepatomegaly [measurement only in sternal line (STL)].

Spleen size was available in only 43.3% of patients (13/30) and no age-adjusted splenomegaly was observed.

Intestinal wall thickness was measured in only 50% of cases (44% in the STEC-HUS group and 80% in the cHUS group). It was increased in only 72.7% of the STEC-HUS patients (8/11 patients) and was measured up to 12 mm. The patients with enlarged intestinal walls all had diarrhea at the timepoint of the ultrasound. In the cHUS patients, it was increased to 4 mm in one patient.

No patient displayed ascites or pleural effusion during the US examination (Table 4).

**Table 4:** Other US findings in patients with STEC-HUS or cHUS.

		STEC-HUS		cHUS	
		Evaluable patients		Evaluable patients	
		*n* (%)		*n* (%)	
Echogenicity, *n* (%)	Very high	22 (88%)	12 (54%)	4 (80%)	2 (50%)
	High		6 (27%)		1 (25%)
	Normal		4 (18%)		1 (25%)
Liver (STL) (cm)	Median	13 (52%)	8	4 (80%)	9
	Mean (standard deviation)		8 (1.8)		9.3 (3)
Spleen (cm)	Median	10 (40%)	7	3 (60%)	8
	Mean (standard deviation)		8 (1.9)		8.3 (1.5)
Intestinal wall (mm)	Median	11 (44%)	6	4 (80%)	2
	Mean (standard deviation)		6.4 (3.7)		2.5 (1)

## Discussion

We retrospectively analyzed a large cohort of children with STEC-HUS or complement-mediated HUS with a special focus on ultrasound diagnostics. We found prominent roles for RI at disease onset and total kidney size as indicators of the requirement for dialysis in acute kidney failure in STEC-HUS cases. We also demonstrated the normalization of various ultrasound parameters in the further course of the disease in line with the clinical improvement, as all but one case showed a normalization of renal function.

### Study cohort

The predominant proportion of STEC-HUS in this cohort (83%), the most prevalent EHEC serotype being O157, and the genetic distribution of heterozygous pathogenic variants in cHUS all corresponded to published studies on HUS in childhood (5–7, 19). The median age of disease onset in patients with STEC-HUS was lower compared to those with cHUS (2.9 vs. 5.4 years) and there was an almost balanced sex distribution in both groups; both aspects are consistent with recent multicenter studies (7, 34). The proportion of diarrhea symptoms at the onset of the disease in the STEC-HUS patients was slightly lower than the published data (19) but it was still higher than in the cHUS group. This highlights the challenge that diarrhea at disease onset is not a specific sign of the presence of STEC-HUS and justifies the abandonment of the old classification of patients into either diarrhea- or non-diarrhea-associated HUS. Accordingly, as to be expected, C3 levels were higher in the cHUS cohort but less than half of the cases were in line with other studies (35), which makes it difficult to reliably identify a decisive role of the complement system in the early disease phase. In this and other studies (36, 37), temporary complement activation was present in some STEC-HUS cases, making the initial differentiation of HUS types even more difficult. The higher frequency of hypertension in cHUS is most likely due to recurrent damage to the kidney tissue as part of the often wave-like course of cHUS (19). The proportion of children requiring renal replacement therapy for acute renal failure was in accordance with previous studies and was almost the same for both the STEC-HUS and cHUS groups with a frequency ranging between 60% and 68% (19, 38).

### Ultrasound analysis

The typical laboratory constellation is indicative for the diagnosis of all forms of HUS; therefore, a kidney biopsy does not contribute to a decisive gain in clinical knowledge, and the risk of complications is significantly increased in thrombocytopenia. However, the application of ultrasound as a non-invasive tool supports the confirmation of diagnosis and can influence the management of the treatment of HUS, which is evident in our analysis.

One key result of our data is the significantly increased total kidney size in children with both HUS forms at disease onset. The subgroup of children with STEC-HUS that required acute dialysis demonstrated significantly larger total kidney sizes at disease onset compared to those that did not. Although the few available publications on ultrasound examinations in HUS show an increase in renal size above the normal range, no further quantification of the extent of the increase in size was undertaken (26, 38, 39). Therefore, the importance of kidney size in estimating acute dialysis requirements in STEC-HUS has not yet been described in the literature. The relative value for kidney size of 141% shown in the ROC analysis can therefore serve as a guide for early decision-making regarding the need for dialysis in patients with STEC-HUS and dynamic acute renal failure. The children with cHUS also showed similarly increased kidney sizes at disease onset. Further prospective studies in cHUS are required to evaluate whether kidney size is also more increased in children with cHUS who require acute dialysis as the small number of cHUS cases in our study was not sufficient for reliable analysis.

Another key result of our data is the role of significantly increased RI values, as a marker of the ratio between systolic peak flow velocity and end-diastolic flow velocity in the renal artery. In both the STEC-HUS and cHUS groups, we found an increased RI value in the renal artery compared to normal values, in particular as an expression of reduced end-diastolic flow velocity caused by thrombotic renal microangiopathy. The RI values in STEC-HUS patients at disease onset were comparable in the subcohorts (i.e., those who did or did not require dialysis) and therefore were not reliable for predicting dialysis indication alone, as was also demonstrated in the ROC analysis. The study by Reising et al. (38) confirms our study results in relation to elevated RI values in an adult cohort and, additionally, demonstrated higher RI values in STEC-HUS patients compared to cHUS patients at disease onset, which might be due to the different pathophysiologies, resulting in a more undulating clinical course in cHUS compared to the peak-like course in STEC-HUS. However, considering the small cHUS cohort in our study, our data did not demonstrate statistically significant numerical differences in elevated RI values in the STEC-HUS group compared to the cHUS group. In general, the RI values in all adult subcohorts with HUS are lower compared to our data in children, which is explained by the physiologically lower RI values in healthy adults compared to children (31, 38). While the RI value in adults was statistically significantly higher in patients with STEC-HUS who required dialysis compared to patients who did not, our data showed no significant differences in our cohort of children with HUS and, overall, significantly higher RI values (38).

A classification model using both US parameters (total kidney size and RI) sequentially might be of clinical use for predicting dialysis requirements in patients with STEC-HUS with high sensitivity and specificity. As part of a decision tree, all children with STEC-HUS who require dialysis can be recognized from a relative kidney size above 130% due to the high sensitivity, however, specificity is low. Therefore, an additional RI measurement is required in this subgroup in the next step and RI values display a high specificity to distinguish between dialysis-dependent (RI > 1.0) or non-dependent HUS (RI < 1.0) (Figure 4).

Total kidney size and RI of the renal artery decreased significantly at the time of discharge and normalized at follow-up after 1 year in line with the clinical improvement. All but one case showed a normalization of renal function. The subgroup of patients with STEC-HUS who required dialysis at disease onset still displayed increased kidney size at the time of discharge, emphasizing its suitability for progress monitoring.

Nearly all of our patients with STEC-HUS and cHUS demonstrated increased renal cortical echogenicity at disease onset, similar to previous results (26, 28), but without correlation with dialysis, GFR, or uremic acid.

The significant increase in RI is more specific for the presence of HUS as opposed to other causes of acute renal failure, as demonstrated in Reising's data (38). In contrast, increased kidney size is not specific for HUS as increased size and cortical echogenicity are also hallmarks of other forms of acute kidney injury just as decreased kidney sizes with reduced corticomedullary differentiation are typical for chronic kidney failure (23). Extrarenal ultrasound abnormalities such as hepatosplenomegaly, ascites, or thickening of the intestinal wall were common in the STEC-HUS patients and can be helpful in assessing further organ involvement and fluid balance.

Renal ultrasound as a non-invasive technique is an indispensable and valuable diagnostic tool to assess individual kidney involvement and the need for replacement therapy in STEC-HUS and cHUS patients but does not the reliable ability to distinguish between these different types. Ultrasound examination might be especially crucial in clinical cases when the diagnosis of HUS and the pathophysiological variant might be pending. New emerging US technologies such as SMI, which enables detailed tissue perfusion with pinpoint accuracy, have the potential to expand the possibilities for diagnostics and therapy control in HUS and in acute renal failure in general (40).

### Limitations

The main limitation of this study is its retrospective design. The other main limitation is the small number of patients with complement-mediated HUS. Thus, the analysis of and conclusions drawn from this subcohort were significantly more limited compared to the larger cohort of children with STEC-HUS. A further disadvantage with regard to the decision tree also results from the small number of observations (*n* = 14). This meant that we were unable to divide our data into training and test datasets. It was, therefore, not possible to calculate the expected out-of-sample performance of our decision tree. The sensitivity, specificity, and balanced accuracy refer exclusively to the training data. An evaluation of the tree is possible through further data collection and subsequent testing of the tree. Another relevant limitation is the fact that in a considerable number of patients, clinical data at the time of discharge and in particular for the 1-year follow-up could not be obtained due to retrospective data collection and loss of follow-up. Therefore, analysis regarding the use of ultrasound in the clinical course is limited. In addition, most of the ultrasound examinations at disease onset were performed in emergency settings by different pediatricians with different ultrasound devices. Although only children with adequate US images were included, image quality was variable and not all parameters collected in this study were documented in every US examination. However, the present cohort of children with HUS included a significant number of patients with an outstanding US examination compared to previous clinical studies.

## Conclusion

Increases in the US parameters of total kidney size, RI, and renal cortical echogenicity were present at disease onset in both HUS forms and normalized in the clinical course in line with the clinical improvement. In particular, kidney size at disease onset appears to be larger in patients with STEC-HUS who required dialysis compared to patients who did not. Therefore, total kidney size in relation to the normal value may be useful in the clinical assessment of HUS at disease onset together with RI. Future prospective studies are required to assess the diagnostic capability of emerging ultrasound technologies in evaluating different forms of HUS in children and predicting the severity of AKI and need for dialysis.

## Data Availability

The datasets presented in this study can be found in online repositories. The names of the repository/repositories and accession number(s) can be found in the article/Supplementary Material.

## References

[1] Jokiranta TS. HUS and atypical HUS. Blood. (2017) 129(21):2847–56. 10.1182/blood-2016-11-709865 28416508 PMC5445567

[2] Bagga A Khandelwal P Mishra K Thergaonkar R Vasudevan A Sharma J Hemolytic uremic syndrome in a developing country: consensus guidelines. Pediatr Nephrol. (2019) 34(8):1465–82. 10.1007/s00467-019-04233-7 30989342

[3] Igarashi T Ito S Sako M Saitoh A Hataya H Mizuguchi M Guidelines for the management and investigation of hemolytic uremic syndrome. Clin Exp Nephrol. (2014) 18(4):525–57. 10.1007/s10157-014-0995-9 25099085

[4] Scully M Hunt BJ Benjamin S Liesner R Rose P Peyvandi F Guidelines on the diagnosis and management of thrombotic thrombocytopenic purpura and other thrombotic microangiopathies. Br J Haematol. (2012) 158(3):323–35. 10.1111/j.1365-2141.2012.09167.x 22624596

[5] Joseph A Cointe A Mariani Kurkdjian P Rafat C Hertig A. Shiga toxin-associated hemolytic uremic syndrome: a narrative review. Toxins (Basel). (2020) 12(2):67. 10.3390/toxins12020067 31973203 PMC7076748

[6] Spinale JM Ruebner RL Copelovitch L Kaplan BS. Long-term outcomes of Shiga toxin hemolytic uremic syndrome. Pediatr Nephrol. (2013) 28(11):2097–105. 10.1007/s00467-012-2383-6 23288350

[7] Ylinen E Salmenlinna S Halkilahti J Jahnukainen T Korhonen L Virkkala T Hemolytic uremic syndrome caused by Shiga toxin-producing Escherichia coli in children: incidence, risk factors, and clinical outcome. Pediatr Nephrol. (2020) 35(9):1749–59. 10.1007/s00467-020-04560-0 32323005 PMC7385025

[8] Dücker C Dautel P Wagner K Przewozna J Oehlerking S Repenthin J Clinical symptoms, treatment and outcome of EHEC and EHEC-HUS patients treated as in-patients. Dtsch Med Wochenschr. (2011) 136(36):1770–6. 10.1055/s-0031-1286099 21882131

[9] Frank C Werber D Cramer JP Askar M Faber M an der Heiden M Epidemic profile of Shiga-toxin-producing Escherichia coli O104:H4 outbreak in Germany. N Engl J Med. (2011) 365(19):1771–80. 10.1056/NEJMoa1106483 21696328

[10] Nester CM Barbour T de Cordoba SR Dragon-Durey MA Fremeaux-Bacchi V Goodship TH Atypical aHUS: state of the art. Mol Immunol. (2015) 67(1):31–42. 10.1016/j.molimm.2015.03.246 25843230

[11] Çakar N Ozcakar ZB Ozaltin F Koyun M Celikel Acar B Bahat E Atypical hemolytic uremic syndrome in children aged <2 years. Nephron. (2018) 139(3):211–8. 10.1159/000487609 29533929

[12] Zimmerhackl LB Besbas N Jungraithmayr T van de Kar N Karch H Karpman D Epidemiology, clinical presentation, and pathophysiology of atypical and recurrent hemolytic uremic syndrome. Semin Thromb Hemost. (2006) 32(2):113–20. 10.1055/s-2006-939767 16575686

[13] Lemaire M Frémeaux-Bacchi V Schaefer F Choi M Tang WH Le Quintrec M Recessive mutations in DGKE cause atypical hemolytic-uremic syndrome. Nat Genet. (2013) 45(5):531–6. 10.1038/ng.2590 23542698 PMC3719402

[14] Sharma AP Greenberg CR Prasad AN Prasad C. Hemolytic uremic syndrome (HUS) secondary to cobalamin C (cblC) disorder. Pediatr Nephrol. (2007) 22(12):2097–103. 10.1007/s00467-007-0604-1 17874135

[15] Guerra OJL Rodríguez RSG Camacho WJM Ortiz JEP Camacho MAM. Hemolytic uremic syndrome associated with streptococcus pneumoniae in pediatrics: a case series. Rev Paul Pediatr. (2019) 38:e2018065. 10.1590/1984-0462/2020/38/2018065 31778402 PMC6909244

[16] Bayer G von Tokarski F Thoreau B Bauvois A Barbet C Cloarec S Etiology and outcomes of thrombotic microangiopathies. Clin J Am Soc Nephrol. (2019) 14(4):557–66. 10.2215/CJN.11470918 30862697 PMC6450353

[17] Garg AX Suri RS Barrowman N Rehman F Matsell D Rosas-Arellano MP Long-term renal prognosis of diarrhea-associated hemolytic uremic syndrome: a systematic review, meta-analysis, and meta-regression. JAMA. (2003) 290(10):1360–70. 10.1001/jama.290.10.1360 12966129

[18] Kavanagh D Goodship TH Richards A. Atypical hemolytic uremic syndrome. Semin Nephrol. (2013) 33(6):508–30. 10.1016/j.semnephrol.2013.08.003 24161037 PMC3863953

[19] Loirat C Fakhouri F Ariceta G Besbas N Bitzan M Bjerre A Frémeaux-Bacchi V; HUS international. An international consensus approach to the management of atypical hemolytic uremic syndrome in children. Pediatr Nephrol. (2016) 31(1):15–39. 10.1007/s00467-015-3076-8 25859752

[20] Avila Bernabeu AI Cavero Escribano T Cao Vilarino M. Atypical hemolytic uremic syndrome: new challenges in the complement blockage era. Nephron. (2020) 144(11):537–49. 10.1159/000508920 32950988

[21] Bush WH Jr Amis ES Jr Bigongiari LR Bluth EI Choyke PL Fritzsche P Radiologic investigation of causes of renal failure. American college of radiology. ACR appropriateness criteria. Radiology. (2000) 215(Suppl):713–20. 11037490

[22] O'Neill WC. Sonographic evaluation of renal failure. Am J Kidney Dis. (2000) 35(6):1021–38. 10.1016/s0272-6386(00)70036-9 10845813

[23] Ozmen CA Akin D Bilek SU Bayrak AH Senturk S Nazaroglu H. Ultrasound as a diagnostic tool to differentiate acute from chronic renal failure. Clin Nephrol. (2010) 74(1):46–52. 20557866

[24] Patriquin HB O'Regan S Robitaille P Paltiel H. Hemolytic-uremic syndrome: intrarenal arterial Doppler patterns as a useful guide to therapy. Radiology. (1989) 172(3):625–8. 10.1148/radiology.172.3.2672090 2672090

[25] Scholbach TM. Changes of renal flow volume in the hemolytic-uremic syndrome–color Doppler sonographic investigations. Pediatr Nephrol. (2001) 16(8):644–7. 10.1007/s004670100638 11519894

[26] Glatstein M Miller E Garcia-Bournissen F Scolnik D. Timing and utility of ultrasound in diarrhea-associated hemolytic uremic syndrome: 7-year experience of a large tertiary care hospital. Clin Pediatr (Phila). (2010) 49(5):418–21. 10.1177/0009922809342582 20075028

[27] Kenney PJ Brinsko RE Patel DV Spitzer RE Farrar FM. Sonography of the kidneys in hemolytic uremic syndrome. Invest Radiol. (1986) 21(7):547–50. 10.1097/00004424-198607000-00006 3525450

[28] Choyke PL Grant EG Hoffer FA Tina L Korec S. Cortical echogenicity in the hemolytic uremic syndrome: clinical correlation. J Ultrasound Med. (1988) 7(8):439–42. 10.7863/jum.1988.7.8.439 3047422

[29] Deeg K Hofmann V Hoyer PF. Ultraschalldiagnostik in Pädiatrie und Kinderchirurgie: Lehrbuch und Atlas. Stuttgart: Georg Thieme Verlag (2018).

[30] Obrycki L Sarnecki J Lichosik M Sopińska M Placzyńska M Stańczyk M Kidney length normative values in children aged 0-19 years—a multicenter study. Pediatr Nephrol. (2022) 37(5):1075–85. 10.1007/s00467-021-05303-5 34657197 PMC9023417

[31] Kuzmić AC Brkljacić B Ivanković D Galesić K. Doppler sonographic renal resistance index in healthy children. Eur Radiol. (2000) 10(10):1644–8. 10.1007/s003300000466 11044940

[32] Deeg KH Wörle K Wolf A. Doppler sonographic estimation of normal values for flow velocity and resistance indices in renal arteries of healthy infants. Ultraschall Med. (2003) 24(5):312–22. 10.1055/s-2003-42913 14562209

[33] Armitage P Berry G. Statistical Methods in Medical Research. 3 edn. Oxford: Blackwell Scientific Publications (1994).

[34] Fremeaux-Bacchi V Fakhouri F Garnier A Bienaimé F Dragon-Durey MA Ngo S Genetics and outcome of atypical hemolytic uremic syndrome: a nationwide French series comparing children and adults. Clin J Am Soc Nephrol. (2013) 8(4):554–62. 10.2215/CJN.04760512 23307876 PMC3613948

[35] Hofer J Janecke AR Zimmerhackl LB Riedl M Rosales A Giner T Complement factor H-related protein 1 deficiency and factor H antibodies in pediatric patients with atypical hemolytic uremic syndrome. Clin J Am Soc Nephrol. (2013) 8(3):407–15. 10.2215/CJN.01260212 23243267 PMC3586960

[36] Thurman JM Marians R Emlen W Wood S Smith C Akana H Alternative pathway of complement in children with diarrhea-associated hemolytic uremic syndrome. Clin J Am Soc Nephrol. (2009) 4(12):1920–4. 10.2215/CJN.02730409 19820137 PMC2798880

[37] Ahlenstiel-Grunow T Hachmeister S Bange FC Wehling C Kirschfink M Bergmann C Systemic complement activation and complement gene analysis in enterohaemorrhagic Escherichia coli-associated paediatric haemolytic uraemic syndrome. Nephrol Dial Transplant. (2016) 31(7):1114–21. 10.1093/ndt/gfw078 27190382

[38] Reising A Hafer C Hiss M Kielstein JT Menne J Gueler F Ultrasound findings in EHEC-associated hemolytic-uremic syndrome and their clinical relevance. Int Urol Nephrol. (2016) 48(4):561–70. 10.1007/s11255-015-1194-7 26759326

[39] Lemmer A Bergmann K Walch R Endert G. Doppler ultrasound studies of long-term follow-up of children with hemolytic-uremic syndrome. Ultraschall Med. (1995) 16(3):127–31. 10.1055/s-2007-1003169 7667621

[40] Armaly Z Abu-Rahme M Kinaneh S Hijazi B Habbasshi N Artul S. An innovative ultrasound technique for early detection of kidney dysfunction: superb microvascular imaging as a reference standard. J Clin Med. (2022) 11(4):925. 10.3390/jcm11040925 35207202 PMC8878179

