# Supplementary material for: Ultrasound analysis of different forms of hemolytic uremic syndrome in children
Source: Front Pediatr. 2024 Oct 23;12:1433812. doi: 10.3389/fped.2024.1433812 (PMC11537852; doi:10.3389/fped.2024.1433812)
Supplement: Supplementary file 2 [file Image1.pdf]

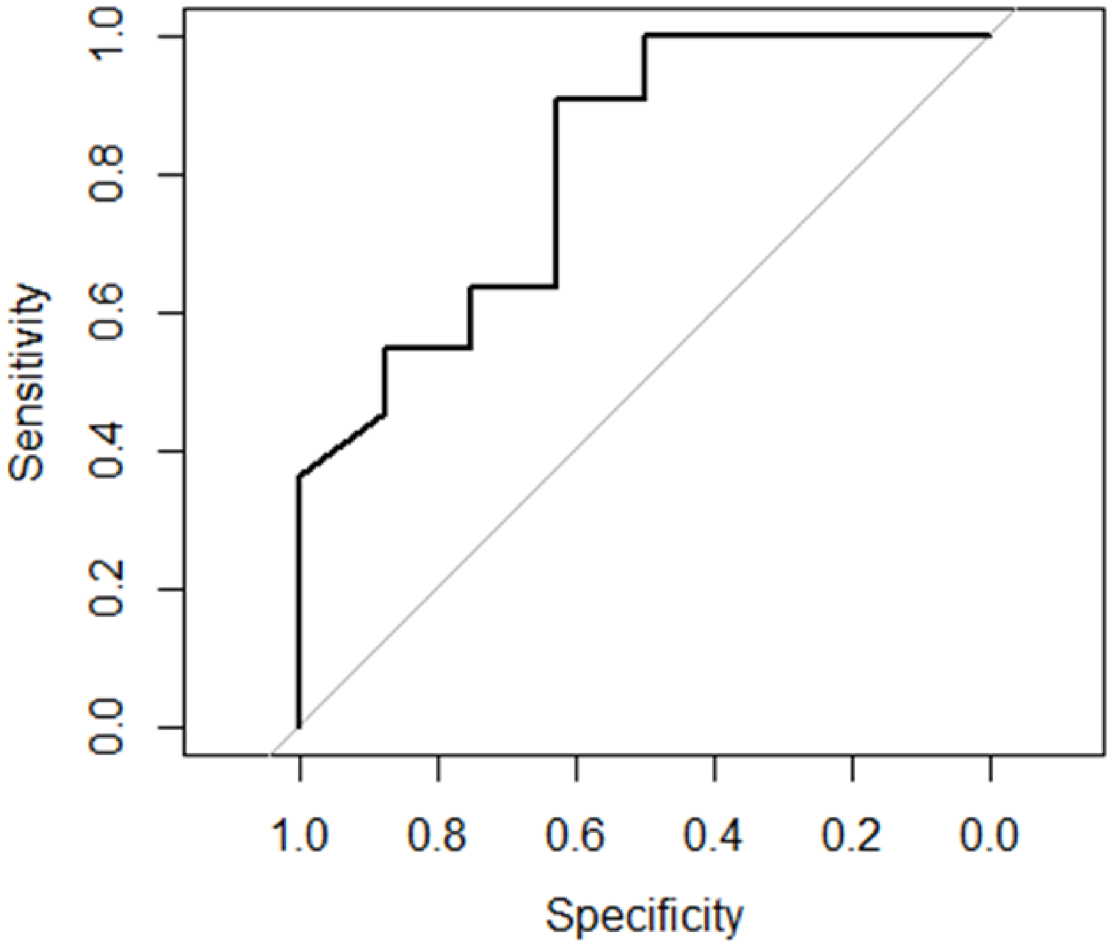

Supplementary Figure 1a – ROC curve of classification of dialysis requirement using the predictor kidney size.

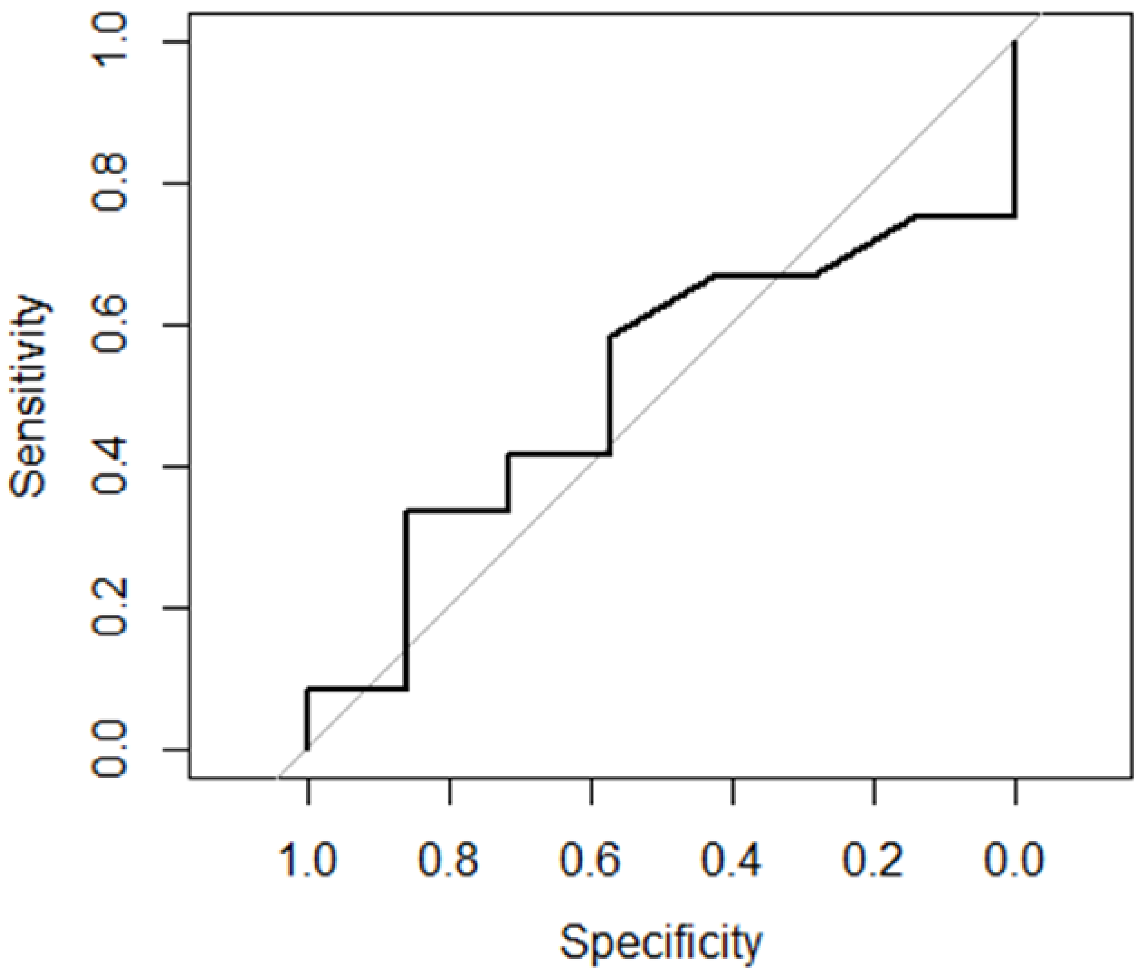

Supplementary Figure 1b – ROC curve of classification of dialysis requirement using the predictor RI value.
